# Reframing ‘chronic conditions’ in adolescent health: why terminology matters for person-centred and equitable care

**DOI:** 10.1080/16549716.2026.2634465

**Published:** 2026-03-03

**Authors:** Talitha Crowley

**Affiliations:** School of Nursing, University of the Western Cape, Cape Town, South Africa

**Keywords:** Adolescents, chronic conditions, health systems, long-term health conditions, person-centred care

## Abstract

The terms *chronic health conditions* and *long-term health conditions* are often used interchangeably, yet they carry distinct conceptual emphases. Chronic health conditions typically prioritise ongoing medical management reflecting a predominantly biomedical and adult-centred care logic. In contrast, long-term health conditions foreground the enduring consequences of illness for an individual’s development and wellbeing across the life course. This debate examines the implications of these terms, particularly for adolescent health, arguing that persistent reliance on adult-centred biomedical framings obscures developmental, relational and contextual dimensions of care. Intentional reframing of terminology in policy, research and practice can support adolescent-centred, developmentally appropriate approaches that extend beyond disease control to encompass physical, psychological and social wellbeing. Such a shift has implications for the design of interventions, service delivery and policy frameworks, enabling healthcare systems to better support adolescents’ needs and their transition to adulthood. While adolescence provides a critical lens, this reframing holds relevance across the life course by strengthening person-centred approaches to care.

## Paper Context

- **Main findings:** The paper demonstrates that the terms *chronic conditions* and *long-term health conditions* are not conceptually equivalent and that their use carries important implications for how adolescent health is framed in policy, research and practice.
- **Added knowledge:** This paper clarifies the conceptual distinctions between *chronic conditions* and *long-term health conditions*, showing how terminology shapes adolescent health framing, identity, inclusion of mental health and models of care.
- **Global health impact for policy and action:** Adopting terminology that reflects developmental and contextual realities can support more equitable, person-centred policies and service delivery models. Such reframing has the potential to improve adolescent wellbeing and contribute to progress towards the Sustainable Development Goals.

## Background

Adolescence, typically defined as ages 10–19 years, and sometimes extended to 24 years, is a critical period of biological, psychological and social development [1,2]. Optimal support during this stage fosters resilience, wellbeing and the capabilities required for healthy transitions into adulthood. Nearly half of the world’s adolescents live in multi-burden settings, facing intersecting environmental, social and systemic challenges, including climate change, pandemics, urbanisation, conflict and exposure to potentially harmful commercial and digital influences [1]. These conditions compound developmental vulnerability and increase the complexity of supporting health and wellbeing during adolescence.

Adolescents worldwide experience a broad spectrum of health conditions, including communicable diseases (e.g. HIV, tuberculosis, malaria), non-communicable diseases (e.g. obesity-related conditions, diabetes, hypertension, asthma and cancer), nutritional deficiencies, maternal-related conditions associated with adolescent pregnancy and injuries [1,3]. Many of these conditions have enduring physical and psychosocial consequences that intersect with key developmental tasks. The growing prevalence of obesity and mental health disorders exemplifies the multidimensional nature of adolescent health challenges and underscores the need for integrated, developmentally appropriate responses [1].

## Terminology matters

The terms *chronic disease* and *long-term health condition* are often used interchangeably in the literature, policy and clinical practice. Historically, chronic diseases have been defined as ‘conditions that last 1 year or more and require ongoing medical attention or limit activities of daily living or both.’ [4] This framing has been largely shaped by adult health priorities, emphasising biomedical management, lifestyle-related risk factors and long-term disease control. Similarly, the World Health Organization commonly aligns chronic diseases with non-communicable diseases, identifying cardiovascular disease, respiratory disease, diabetes and cancer as leading contributors to adult mortality [5]. Although the term *chronic disease* has been applied across the life course to describe incurable conditions of prolonged duration, this undifferentiated use risks obscuring developmentally specific needs, producing conceptual blind spots at the developmental extremes.

While the term *chronic disease* supports epidemiological surveillance and clinical management, it inadequately captures the multimorbid, developmental and psychosocial dimensions of adolescent health. Ongoing debate reflects uncertainty about whether mental health conditions should be included within this category. For example, Spencer et al. define ‘chronic physical conditions’ as long-term controllable but not curable conditions, thereby excluding mental health [6]. In contrast, Goodman et al. highlight the heterogeneity of existing definitions and the resulting challenges for consistent measurement, advocating for the more inclusive term ‘chronic conditions’, encompassing both physical and mental health [7].

Building on a developmental perspective, Stein et al. argue that identifying chronic conditions in children according to their consequences rather than diagnosis better supports long-term care planning and developmental outcomes [8]. Reflecting this shift, the term *long-term health conditions* has since been adopted in policy frameworks relating to children, adolescents and youth in the UK, Australia and South Africa [3,9,10]. Westwood and Slemming [3] define long-term health conditions as physical, cognitive or mental conditions characterised by: i) duration of more than 1 year with potential developmental impact; ii) the need for comprehensive, coordinated care; and iii) onset at any point from birth onward. These conditions encompass a wide spectrum, including intellectual disabilities and congenital abnormalities, psychiatric and neuro-behavioural conditions, allergic (e.g. asthma), autoimmune (e.g. diabetes type 1), cardiovascular (e.g. hypertension), infectious (e.g. HIV), nutritional and oncological disorders.

## Implications for policy and practice

Despite growing recognition of adolescents’ unique developmental needs, adult-centric terminology continues to dominate global health frameworks, shaping surveillance, funding priorities and models of care in ways that obscure psychosocial and developmental considerations. The persistence of *chronic conditions* in flagship reports such as the *Lancet Commission* and the *Global Accelerated Action for the Health of Adolescents* (WHO AA-HA!) guidance reflects a conceptual lag in adolescent health discourse. By contrast, the *South African Child Gauge’s* adoption of *long-term health conditions* exemplifies a more explicitly developmental and adolescent-centred framing. Such terminological inconsistencies influence measurement, reporting and funding decisions, including whether mental health comorbidities are systematically recognised, thereby shaping programme design and evaluation.

The implications of linguistic framing extend beyond semantics. Mol’s *Logic of Care* (2008) illustrates how language reflects underlying ‘logics’ of health care. A ‘logic of choice’ positions individuals as autonomous managers of their health, whereas a ‘logic of care’ foregrounds interdependence, adaptation and relational support [11]. For adolescents, whose self-management capacities are evolving and contingent on family, peer and health system support, terminology shapes both policy discourse and lived experience. The reframing from ‘HIV-positive adolescents’ to ‘adolescents living with HIV (ALHIV)’ illustrates how language can reduce stigma, support identity development and promote person-centred care. At the same time, disease-focused models of care, while effective in targeted contexts, can inadvertently reinforce fragmented funding and policy priorities, often privileging single-disease programmes such as HIV at the expense of integrated approaches that address co-existing long-term health conditions in adolescence.

Dominant chronic disease framings often assume a stable diagnosis, a predictable disease trajectory and a high degree of individual self-management, with limited attention to the dynamic interaction between biological, social and environmental contexts. In contrast, the term *long-term health conditions* acknowledges ongoing change across developmental stages and life circumstances, thereby supporting a more explicitly person-centred and context-responsive approach to care.

These conceptual distinctions have tangible implications. Approximately 25% of adolescents live with one or more long-term health conditions, with the prevalence of multimorbidity increasing [3]. These adolescents navigate normative developmental tasks alongside condition management and frequently encounter fragmented services [12]. Framing conditions by their long-term consequences rather than diagnosis facilitates more comprehensive and integrated, adolescent-centred care, encompassing self-management support, stigma reduction, mental health, sexual reproductive health, cardiovascular risk factors, multimorbidity and sustained engagement with services.

Adopting a life-course approach to long-term health conditions also supports continuity of care as adolescents transition to adult services and recognises the shared responsibilities of families, communities, educators and health professionals in supporting evolving capacities over time. While adolescence serves as a critical analytic lens that exposes the limitations of chronic disease logic most clearly, the proposed reframing has broader relevance across the life course, including older adults, by enabling care models that respond to changing support needs at different stages of life.

## Conclusion

Terminology is more than semantics; it shapes philosophy, practice and policy. When left unexamined, language can quietly sustain misaligned systems of care. Using adolescent health as a critical analytic lens, this paper argues that reframing chronic disease as long-term health conditions foregrounds developmental appropriateness, relational care and multisectoral collaboration. More consistent terminology supports accurate measurement, adolescent-responsive care models and integrated service design that extend beyond disease control to encompass lived experience and wellbeing. Adolescents should be actively engaged in dialogue about the language used to describe their health and care, as their perspectives are central to reducing stigma, supporting identity development and ensuring that interventions reflect lived realities. While adolescence exposes the limitations of dominant chronic disease framings most clearly, the proposed reframing has relevance across the life course, offering a foundation for more person-centred, equitable care for individuals at all stages of life.
